# Induced Pluripotent Stem Cell-Derived Organoids: Their Implication in COVID-19 Modeling

**DOI:** 10.3390/ijms24043459

**Published:** 2023-02-09

**Authors:** Mária Csöbönyeiová, Martin Klein, Marcela Kuniaková, Ivan Varga, Ľuboš Danišovič

**Affiliations:** 1 Regenmed Ltd., Medena 29, 811 01 Bratislava, Slovakia; 2 Institute of Histology and Embryology, Faculty of Medicine, Comenius University, Sasinkova 4, 811 08 Bratislava, Slovakia; 3 Institute of Medical Biology, Genetics and Clinical Genetics, Faculty of Medicine, Comenius University, Sasinkova 4, 811 08 Bratislava, Slovakia

**Keywords:** COVID-19, disease modeling, iPSCs, organoids

## Abstract

The outbreak of coronavirus disease 2019 (COVID-19) caused by severe acute respiratory syndrome coronavirus 2 (SARS-CoV-2) has become a significant global health issue. This novel virus’s high morbidity and mortality rates have prompted the scientific community to quickly find the best COVID-19 model to investigate all pathological processes underlining its activity and, more importantly, search for optimal drug therapy with minimal toxicity risk. The gold standard in disease modeling involves animal and monolayer culture models; however, these models do not fully reflect the response to human tissues affected by the virus. However, more physiological 3D in vitro culture models, such as spheroids and organoids derived from induced pluripotent stem cells (iPSCs), could serve as promising alternatives. Different iPSC-derived organoids, such as lung, cardiac, brain, intestinal, kidney, liver, nasal, retinal, skin, and pancreatic organoids, have already shown immense potential in COVID-19 modeling. In the present comprehensive review article, we summarize the current knowledge on COVID-19 modeling and drug screening using selected iPSC-derived 3D culture models, including lung, brain, intestinal, cardiac, blood vessels, liver, kidney, and inner ear organoids. Undoubtedly, according to reviewed studies, organoids are the state-of-the-art approach to COVID-19 modeling.

## 1. Introduction

The coronavirus disease 2019 (COVID-19) was classified as a public health emergency of international concern and declared a global pandemic by the World Health Organization (WHO) on 11 March 2020. As of late December 2022, over 6,6 million deaths have been reported worldwide [1]. It is well-known that, besides the widely recognized COVID-19 symptoms related to the respiratory tract, such as pneumonia and acute respiratory distress syndrome (ARDS), the virus also affects organs other than the lungs, including the heart, brain, kidneys, intestine, and pancreas, and can even cause systemic injuries of multiple organs [2]. The most common cardiovascular complications are myocardial injuries, arrhythmia (ventricular tachycardia and atrial fibrillation), coagulopathies, and heart failure [3]. The symptoms of gastrointestinal tract infections often present as diarrhea, vomiting, anorexia, nausea, abdominal pain, and gastrointestinal bleeding [4,5]. Mild neurological symptoms, such as headaches, myalgia, anosmia, dizziness, visual dysfunction, neuralgia, and brain fog are also frequently observed in COVID-19/post-COVID-19 patients. Less commonly observed (but serious) COVID-19-associated conditions include acute necrotizing encephalopathy, encephalitis, ischemic stroke, intracerebral hemorrhage, and Guillain–Barré syndrome [6,7]. As there is a broad spectrum of organ systems that the virus could damage, individuals who suffer from different serious diseases are at a particularly high risk of multiple organ infections or organ failures [8].

Unfortunately, to date, no specific treatment could eradicate SARS-CoV-2; however, the development of vaccines has brought about positive outcomes in mitigating the spread of the virus and decreasing its morbidity and mortality [9,10]. The reasons for such an efficient viral spread and propagation in human cells are not well understood. However, we (almost) know with certainty that angiotensin-converting enzyme 2 (ACE2) is a crucial cell entry site for the spike (S) protein of SARS-CoV-2, and due to the high expression of ACE2 in mammalian cells, the virus can easily cause damage to multiple organs [11,12]. However, many other additional host factors may affect the efficacy and play roles in the COVID-19 pathogenesis, such as auxiliary SARS-CoV-2 receptors (heparan sulfate, Scavenger receptor class B member 1, neuropilin-1), alternatives to ACE2 receptors (e.g., cell surface proteins, tyrosine-protein kinase receptor UFO, low-density lipoprotein receptor class A domain-containing protein 3, C-type lectin domain family 4 member G, interferon-inducible transmembrane proteins), host proteases that mediate the viral entry via the spike (S) protein, priming–transmembrane serine protease 2 (TMPRSS2), transmembrane glycoprotein CD147, members of the phosphatidylinositol 3-kinase (PI3K) pathway, some transcriptional factors, and histone-modifying enzymes [13,14,15]. Lysosomal cysteine protease cathepsin L was also found to be a key factor in SARS-CoV-2 infection and a promising therapeutic target [16]. Once the virus enters the cell, its rapid replication can cause cell apoptosis, causing the release of pro-inflammatory cytokines and chemokines, leading to macrophage activation syndrome and resulting in the so-called “cytokine storm”, which can potentially damage the tissue. This process is evident in severely affected patients who require intensive care treatments, whereas their blood tests often display significantly higher levels of interleukin-6 (IL-6), interleukin-10 (IL-10), and tumor necrosis factor-α (TNFα). Other pathological mechanisms caused by the virus involve the activation of the inflammasome and the following production of active mature interleukin-1β (IL-1β) as a mediator of fever, inflammation, and fibrosis [17,18].

Following the facts mentioned above, the preclinical research studies focused on SARS-CoV-2 pathology, host–virus interaction, replication kinetics, and infection inhibition, represent key factors for subsequent clinical trials oriented on COVID-19 therapy.

Several animal models susceptible to the virus, such as cats, ferrets, golden hamsters, non-human primate models (rhesus macaques, cynomolgus macaques), and transgenic mice expressing ACE2 receptors, are being used as COVID-19 models. However, they often fail to mimic the human physiological environment due to differences in the immune system between species [19,20,21,22,23,24,25]. Moreover, the use of animal models is expensive and time-consuming, which is definitively undesirable in pandemic times when preclinical studies must run fast and effectively. Widely used monolayer culture models are less time-consuming and more cost-friendly than animal models. Still, their significant disadvantage is the lack of complex 3D cellular architecture; therefore, they cannot acquire the desired phenotype [26]. The mentioned issues have led to researchers developing more advanced humanized in vitro 3D multicellular models. These include spheroids and organoids that physiologically resemble organ structures and may fill the gap between 2D cell cultures and animal models. Furthermore, 3D models derived from induced pluripotent stem cells (iPSCs) possess several advantages over those derived from embryonic stem cells (ESCs) or multipotent adult stem cells (ASCs), such as patient-specificity, the secession of ethical issues, and reduced immunogenicity. These iPSC-derived “organs-in- a-dish” are already valuable tools for studying infectious diseases caused by viruses, such as the hepatitis B virus [27], hepatitis C virus [28], Zika virus [29], herpes simplex virus-1 [30], and rotaviruses [31]; more recently, iPSC-derived organoids have also proven their essential role in COVID-19 modeling [32,33,34].

## 2. iPSC-Derived Organoids in COVID-19 Modeling

iPSC-based disease modeling has proven to be a powerful instrument for biomedical research and personalized regenerative medicine, offering an outstanding opportunity to study various disease pathophysiology types and build platforms for high-throughput drug discovery and drug toxicity screening. iPSC-derived organoids represent 3D in vitro tissue models containing multiple organ-specific cell lines that closely simulate the essential aspects of organ structures and functions and are capable of cell–cell and cell–extracellular matrix (ECM) interactions. Generally, the ECM in which the cells are embedded serves as the basal lamina for tissue culture and supports the development of 3D tissue organization. Moreover, fully differentiated organoids can be further passaged and expanded. Such properties enable the investigation of multiple host–pathogen interactions involved in the virus infection process, making iPSC-derived organoids appropriate candidates for COVID-19 modeling [35,36].

### 2.1. iPSC-Derived Airway Organoids

**Figure 1:**
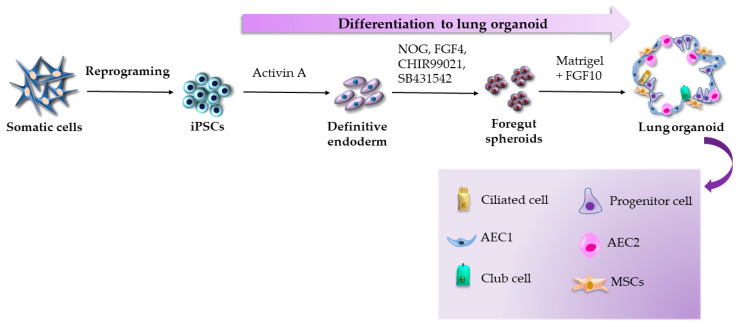
Generation of lung organoids according to the protocol by Dye et al. [43].

Early protocols for airway organoid generations were based on the use of human embryonic stem cells [37,38], primary lung progenitor cells [39,40], adult human alveolar type 2 cells [41], and transformed lung cancer cells [42]; however, the revolutionary iPSC technology brought about the opportunity to create iPSC-derived airway organoids, speeding up their generation and variety. Dye et al. were the first to describe the iPSC-derived lung organoid differentiation protocol, which was based on the simultaneous stimulation and inhibition of different developmental signaling pathways (FGF and HH pathways) by growth factors and small molecules, resembling the processes taking place during normal embryogenesis [43]. First, the authors differentiated the iPSCs into the endoderm by their cultivation in a medium containing activin A. The following step involved the differentiation of the definitive endoderm into the anterior foregut spheroids in a medium supplemented by noggin (NOG), FGF4, CHIR99021, and SB431542. Finally, the foregut spheroids were embedded in Matrigel to maintain the 3D microenvironment and were cultivated in basal medium with the addition of FGF10, an essential factor for branching morphogenesis and maintenance of lung tissue homeostasis. Under the mentioned conditions, the researchers could expand and passage the lung organoids for over 100 days. After two months of cultivation, organoids possessed proximal airway-like structures surrounded by mesenchyme and expressed markers specific for epithelial cells, including those of basal cells, columnar ciliated cells, and club cells. In addition, lung organoids possessed distal epithelial cells as bipotent alveolar progenitor cells, typically present in the fetal lung (Figure 1). From that time, numerous 3D multicellular lung organoids were differentiated from iPSCs [44,45,46,47,48] and have been used to model several upper and even lower respiratory tract diseases, including pulmonary fibrosis [49,50,51], congenital disorders [52,53,54,55], and neonatal respiratory distress syndrome [56]. Most recently, a few research groups developed iPSC-derived lung organoids to model COVID-19 pneumonia [57,58,59].

For instance, Tiwari et al. [57] generated iPSC-derived lung organoids together with iPSC-derived cerebral organoids to study tissue-specific SARS-CoV-2 infection and host response. The lung organoids were differentiated from iPSCs with minor modifications according to the stepwise protocol published by Leibel et al. [48] involving the differentiation of iPSCs into the definitive endoderm (medium supplemented with activin A, RPMI, and FBS), followed by its differentiation into the anterior foregut endoderm/spheroids (basal medium supplemented with SB431542, noggin, SAG, FGF4, and CHIR99021), and finally, lung organoids (basal medium supplemented with FGF7, FGF10, CHIR99021, EGF, ATRA, and VEGF/PIGF). By day 60, the organoids consisted of well-developed epithelium, composed of diverse cells typical for proximal and distal lung regions, such as basal cells, ciliated cells, secretory cells, alveolar type 2 cells (AT2s), and type 1 cells (AT1s). Moreover, Western blot analyses showed significant protein expressions of ACE2 and TMPRSS2 on AT2s. Such lung organoids were subsequently inoculated with SARS-CoV-2 pseudovirus to investigate its entry process and infection. Analyses, such as bright-field imaging, performed 24 h post-infection, revealing the successful incorporation of the virus into the organoids and its co-labeling with ACE2 and TMPRSS2. To test whether the lung organoids could serve as reliable sources of 3D models to screen drugs that could inhibit SARS-CoV-2 infection, the authors treated the organoids with the fusion inhibition peptide (EK1) and TMPRSS2 inhibitor (camostat). As the authors expected, the combination of both compounds notably inhibited the viral infection. These results matched others that were previously published [60,61], thus confirming the usefulness of iPSC-derived organoids for COVID-19 modeling.

An interesting study was published by Spitalieri et al., who tested (on a 3D complex lung organoid model derived from human iPSCs) new immunotherapeutic candidates for COVID-19 treatment involving a tetravalent neutralizing antibody (15033-7) targeting the spike protein and a synthetic peptide homologous to dipeptidyl peptidase-4 (DPP4) receptor for the first time [58]. The authors generated lung organoids by directly differentiating human iPSC colonies, which were obtained by reprogramming human dermal fibroblasts. A serum-free cultivation medium system was used to differentiate iPSCs into lung progenitor cells through three stages (definitive endoderm, anterior foregut endoderm, and lung progenitor cells). Afterward, on day 15, the distal/alveolar differentiations of lung progenitor cells were initiated by their cultivation in an alveolosphere medium supplemented by CHIR99021, rhKGF, dexamethasone, 8-Br-cAMP, and 3-isobutyl-1-methylxanthine. To complete the alveoli maturation, a temporary withdrawal of CHIR99021 was made on days 31–35. The successive passages of alveolospheres in Matrigel droplets achieved their final maturity and prolonged cultivation for up to 105 days. These self-renewing lung organoids consisted of different cell types, with the prevalence of ciliated cells and other structures resembling a fetal lung. The organization of cells was spherical, with well-developed inner cavities corresponding to the morphology of lung alveoli. The quantitative RT-PCR also proved the presence of two AT2 markers mainly expressed in AT2s and non-ciliated bronchial cells. The expression of the ACE2 receptor on the apical surface of AT2s was also distinctly detected. Subsequently, the lung organoids were infected with different SARS-CoV-2 S pseudovirus variants and treated by the 15033-7 antibody or DPP4 peptide. The treatment significantly reduced SARS-CoV-2 infection efficacy in vitro, providing a possible therapeutic option to eliminate virus loading and weaken inflammation and lung damage. Nevertheless, it is important to mention that the study’s major limitation is cell maturation heterogeneity.

Taken together, it is clear that lung organoids derived from iPSCs faithfully recapitulate the pathophysiology and host responses to SARS-CoV-2. Therefore, they are valuable for COVID-19 modeling and allow access to antiviral drug screening. The advantage of lung organoid generation is the ability to construct a model that combines the proximal and distal parts of the airway mimicking the structure of bronchi and distal lungs. As a result, it is feasible to simultaneously study the interactions between infected epithelial cells and stromal components of different airway regions. Another possible way to investigate different airway regions is by the generation of so-called alveolar lung organoids (ALOs), lung airway organoids (AWOs), and bronchial organoids (BCOs), separately. ALOs are solely composed of AT1s, AT2s, and mesenchymal stem cells (MSCs). On the other hand, AWOs consist of various cells comprising the airway epithelium, such as multiciliated cells, basal cells, goblet cells, CC10-secreting club cells, etc. Similarly, the main components of BCOs are cells typical for the bronchial/bronchiolar epithelium, including ciliated cells, basal cells, goblet cells, and club cells [62,63]. In addition, thanks to lung organoids, researchers are able to investigate lung epithelium regeneration after SARS-CoV-2 infection. Moreover, lung organoids are permissive to mass production, cryopreservation, and gene editing and, thus, provide better COVID-19 modeling options over animal models or monolayer cultures.

### 2.2. iPSC-Derived Brain Organoids

**Figure 2:**
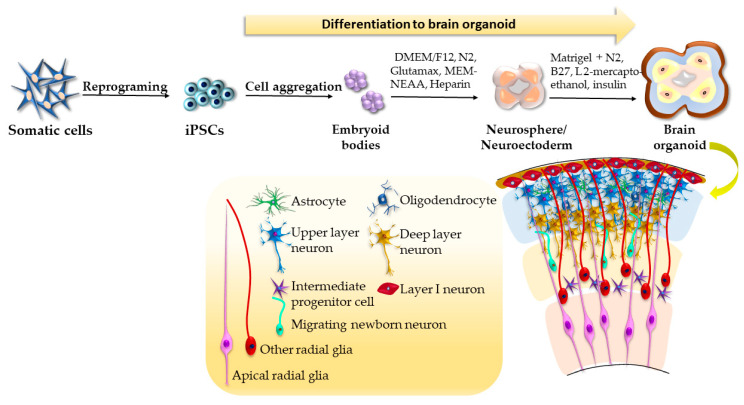
Generation of brain organoids according to the protocol by Lancaster et al. [64].

The in vitro induction of brain organoids recapitulates the general pattering principles along the anterior–posterior and dorsal–ventral axes of the neurodevelopmental process in vivo. The iPSC-derived brain organoids were generated for the first time by a team (Lancaster et al.) using spontaneous differentiation of embryoid bodies (EBs) [64]. The differentiation process involved the generation of neuroectoderm from EBs by their cultivation in neural induction media consisting of DMEM/F12, N2 supplement, GlutaMAX, MEM-NEAA, and heparin. The neuroectodermal tissue was further maintained in 3D culture in differentiation neurobasal media supplemented by N2, B27, L 2-mercaptoethanol, insulin, GlutaMAX, and MEM-NEAA, and embedded in Matrigel droplets, which were later transferred into the spinning bioreactor to provide adequate nutrient support (Figure 2). Brain regions composed of complex heterogeneous tissues, such as the cerebral cortex, midbrain, choroid plexus, immature retina, and meninges were detected by histological and gross morphological analyses in 20–30 days. Furthermore, 30-day brain organoids stained by the marker of the preplate Tbr1 and neuronal marker Map2 displayed the presence of a basal neural layer similar to the embryonic preplate and an apically adjacent region similar to the intermediate zone of the developing brain cortex. However, first brain organoids were highly heterogenous; therefore, the researchers aimed to develop region-specific brain organoids by influencing specific signaling pathways by adding various supplementing factors into the induction medium. For instance, it was found that neuroepithelial cells can be rapidly and efficiently generated from ESCs/iPSCs by dual SMAD inhibition (the presence of dorsomorphin and SB431542) [65,66]. Their further differentiation in a region-specific progenitor can be achieved by the presence of pattering signals of the anterior–posterior (Wnt inhibitors, insulin-like growth factors) or dorsal–ventral axes (ROCK inhibitor, TGFβ inhibitor, Wnt activators, sonic hedgehog agonist, FGF8, CHIR99021) [67,68,69].

Cellular self-organization during organoid differentiation allows the generation of complex brain anatomic regions resembling the dorsal cortex, ventral forebrain, retina, hippocampus, hypothalamus, choroid plexus, and midbrain–hindbrain boundary. These iPSC-derived brain organoids have been used to model various neurological pathologies, such as the toxicity effects of the Zika virus [68,70], sporadic Alzheimer’s disease [71], neurodevelopmental disease [72,73,74], microglia-mediated neuroinflammation [75], and (most recently) the neurotoxic effects of SARS-CoV-2 [76,77,78,79]. The generation of region-specific iPSC-derived brain organoids that closely parallel the neural epithelium has allowed researchers to investigate the mechanisms of SARS-CoV-2 disrupting the hematoencephalic barrier and infecting the cells of the central nervous system (CNS) [80].

Pellegrini et al. examined the expression patterns of SARS-CoV-2 spike pseudovirions and live SARS-CoV-2 entry factors in brain and choroid plexus organoids derived from iPSCs and found that the primary entry point of the virus was in lipoprotein-expressing choroid plexus epithelial cells, which expressed very high levels of ACE2 as well as TMPRSS2 [78]. Interestingly, a lack of viral entry was detected in air–liquid interphase cerebral organoids, meaning that the neurons and glial cells of cortical regions were not infected. Similar results were published by Jacob et al., who established iPSC-derived region-specific brain organoids and iPSC-derived choroid plexus organoids, infected them with SARS-CoV-2, and found that neurons and astrocytes were very rarely infected [76]. On the other hand, choroid plexus surface lining cells displayed a high infection rate and increased cell death. Nevertheless, the published results mentioned above are contrary to findings observed by Ramani et al. [81] and Song et al. [82], who identified that SARS-CoV-2 targets preferably mature neuronal cell types of iPSC-derived brain organoids. Moreover, Ramani et al. found that bodies of SARS-CoV-2-positive neurons contain the aberrant Tau protein, whose mislocation and dysfunction are typically seen in the early stages of Alzheimer’s disease and other tauopathies. The authors hypothesized that the presence of the aberrant Tau protein might be a neuronal stress reaction upon virus entry, and in addition, it can trigger cell death programs [81]. Bullen et al. studied SARS-CoV-2 on the 3D iPSC-derived BrainSphere organotypic model consisting of different types of neurons (dopaminergic, glutamatergic, and GABAergic neurons), astrocytes, and oligodendrocytes [77]. As the authors expected, ACE2 receptors were expressed in neurons but not TMPRSS2. Moreover, virus particles were found within the bodies and axons of SARS-CoV-2 positive neurons whose presence proves that the virus can infect neural cells, probably contributing to neurological outcomes. The research group of Zhang et al. also challenged the iPSC-derived 3D neurospheres and human brain organoids with SARS-CoV-2. The obtained data from several performed analyses (plaque assay, electron microscopy, immunostaining) displayed many virus particles within infected neurospheres. The authors detected an extensive SARS-CoV-2 antigen in peripheral and deeper regions of 35-day-old brain organoids, concretely in TUJ1- and NESTIN-positive cells [83]. Most recently, the study by Mesci et al. confirmed that SARS-CoV-2 productively replicates and promotes the death of cortical neurons in eight-week-old human iPSC-derived brain cortical organoids. At the same time, seven-day post-infections rapidly decreased the number of excitatory synapses [79]. Furthermore, the authors tested the capability of Sofosbuvir—an antiviral candidate used to inhibit SARS-CoV-2 replication—and found that Sofosbuvir could reduce intracellular viral RNA levels in a dose-response manner and, by this mechanism, significantly decrease viral-induced cell death.

Regarding the controversial results of the studies mentioned above, whether SARS-CoV-2 fully propagates in CNS still needs to be clarified. We speculate that these opposite results may have arisen due to differences in neural differentiation and brain organoid generation protocols or to different experimental conditions between laboratories, including the time of SARS-CoV-2 infection and the multiplicity of infection (MOI). Another issue that is often presented in other organoids is their immaturity. The brain organoids often resample more fetal brains than mature ones, which can lead to different SARS-CoV-2 susceptibilities between various neural cells. Nevertheless, the mentioned studies provide evidence of selective SARS-CoV-2 neurotropism and reinforce the position of brain organoids as a platform to study virus-induced brain dysfunctions and screen for suitable treatment options.

### 2.3. iPSC-Derived Intestinal Organoids

Although respiratory symptoms predominate in COVID-19, the highest expression of ACE2 in the human body occurs in the microvilli of intestinal enterocytes, suggesting that the gastrointestinal tract (GIT) may be a potential entry route of SARS-CoV-2 [84,85]. Therefore, several studies have utilized human iPSC-derived intestinal organoids to study viral tropism and create a possible platform for organ-specific drug testing [86,87].

**Figure 3:**
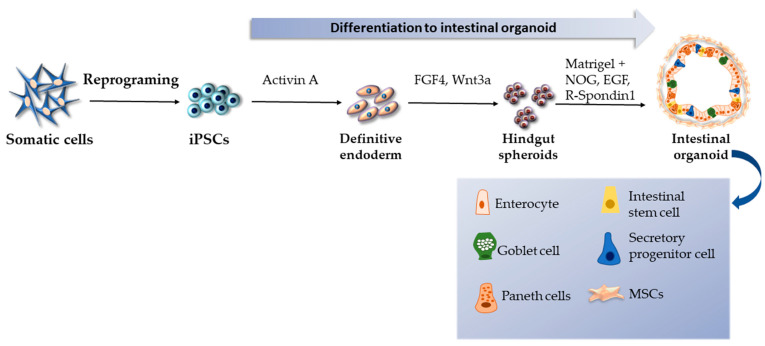
Generation of intestinal organoids according to the protocol by Spence et al. [81].

For the first time, intestinal organoids were differentiated from iPSCs by Spence et al., who established an efficient protocol using a temporal series of growth factors to mimic embryonic intestinal development [88]. The initial step involved the differentiation of iPSCs into cells of the definitive endoderm under the influence of activin A, a nodal-related TGFβ molecule. The next step followed after three days of activin A treatment and involved using Wnt3a and FGF4 to induce hindgut and intestinal specifications. The hindgut spheroids were formed within 2 to 5 days of FGF4 + Wnt3a synergic cultivation. Subsequently, in order to generate 3D intestinal organoids, the floating spheroids were embedded in Matrigel, containing R-spondin 1, noggin, and EGF. Under these specific conditions, the cuboidal epithelium of intestinal spheroids matured into the columnar epithelium (enterocytes, goblet cells, Paneth cells, enteroendocrine cells) with villus-like involutions that protruded into the lumen of the organoid after 28 days (Figure 3). Crypt-like proliferating zones expressing intestinal stem cell markers were also detected. Furthermore, transmission electron microscopy (TEM) showed a well-developed brush border on the apical surface of epithelial cells. According to the GATA factor expression, organoids were composed of a mix of proximal and distal intestinal tissues with secretory and absorptive functionalities, indicating their suitability to model various congenital and infectious gut diseases. Afterward, Múnera et al. reported the achievement of a distal pattering of iPSC-derived colonic organoids via BMP2 stimulation [89].

Later on, further advancements in differentiation techniques and cultivation enabled the generation of iPSC-derived region-specific intestinal organoids with the integrated enteric nervous system and intestinal vasculature [90,91,92]. In the past decade, these small and large intestine organoids (enteroids and colonoids) have provided worthwhile model systems for understanding intestinal pathologies, such as colorectal cancer [93], inflammatory bowel disease [94,95,96], Crohn’s disease [97,98], Hirschsprung disease [99], and ulcerative colitis [100]. Similarly, intestinal organoids are powerful models used to simulate the occurrence of GIT disease mediated by viral infections, such as SARS-CoV-2 [86,87,101,102].

Experiments on intestinal organoids derived from MA104, HEK293 [101], Caco-2, Vero E6, and human ESCs [87,102,103] showed that SARS-CoV-2 productively infects mature enterocytes in a cell-type specific manner (except goblet cells) and that its entry into host cells is facilitated not only by ACE2 but also by TMPRSS2 and TMPRSS4. In addition, Krüger et al. proved that remdesivir and EK1, peptidic pan-coronavirus fusion inhibitors, effectively inhibit SARS-CoV-2 infection and rescue intestinal organoid morphology [102]. Lately, Minthal et al. established a robust method to generate iPSC-derived enteroids and colonoids comprising various regional intestinal epithelia as a valuable tool to study the epithelial response to SARS-CoV-2 infection across cells and tissue types [86]. Intestinal organoids were differentiated from iPSCs through direct differentiation protocols involving definitive endoderm differentiation (StemDiff definitive endoderm kit—days 1 to 3, medium containing dorsomorphin, SB431542—days 3 to 6, and medium containing CHIR99021, rhBMP4, and retinoic acid—days 6 to 14), and final 3D organoid generation in Matrigel droplets with media supplemented with Y27632. Both enteroids and colonoids were subsequently infected by SARS-CoV-2 and displayed an increased number of virus-positive cells with high levels of viral RNA over time. Analyses such as TEM imaging and gene set analyses revealed the presence of characteristic coronaviral particles within enterocytes and the upregulation of apoptosis-related genes, including necroptosis-specific markers. According to the mentioned results, it is evident that SARS-CoV-2 causes cellular stress and inflammatory responses (expression of general pro-inflammatory markers—HAVCR1, PLA2G2A), which may lead to the induction of cell death and related epithelial cell damage in vivo.

As the bottom line, the mentioned studies prove the suitability of iPSC-derived intestinal organoids for SARS-CoV-2 modeling in terms of competency to recapitulate coronavirus infection and simulate human native intestinal epithelium. Moreover, with the use of intestinal organoids, it was also possible to examine the process of intestinal barrier disruption and metabolite transport dysregulation, which are the leading causes of diarrhea in infected patients. However, some authors noticed possible limitations of the experiments, which lie in the incomplete maturity of some epithelial cells resembling fetal-like cells; therefore, further studies are admittedly necessary for revealing all cell-signaling components involved in intestinal SARS-CoV-2 infection. It is also necessary to stress that intestinal organoids are often termed confusingly. Some authors use the abovementioned classification into enteroids and colonoids. However, according to the most recent paper by Han et al., when discussing intestinal organoids, the best approach is to distinguish between small intestinal organoids, colonic organoids, and ileal organoids [62]. Establishing intestinal organoids that mimic different parts of the gut can provide broader SARS-CoV-2 modeling and drug testing options.

### 2.4. iPSC-Derived Liver Organoids

Other common symptoms of patients suffering from COVID-19 include liver enzyme abnormalities and hepatitis contributing to hepatic impairment [104]. Therefore, several research groups used liver organoids to investigate whether liver damage results from the direct viral infection of hepatocytes or a large systemic inflammatory response [105,106].

**Figure 4:**
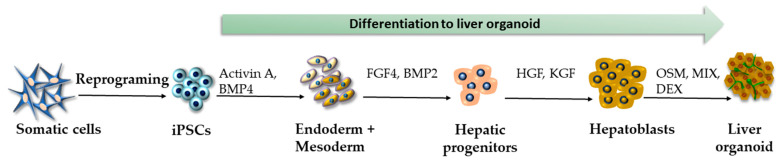
Generation of liver organoids according to the protocol by Wu et al. [110].

In the past, there have been several endeavors to generate liver organoids from iPSCs by different methods resulting in vascularized organoids, cholangiocytes-based organoids, and most recently, “liver-on-a-chip” models [107]. The protocols of iPSC differentiation in liver cells attempt to resemble liver development during embryogenesis, as much as possible, involving posterior foregut endoderm differentiation, which gives rise to hepatic progenitor cells and hepatoblasts. These events are influenced by signaling factors, such as FGF, BMP, hepatocyte growth factor (HGF), and Wnt. The following differentiation toward liver bud and, finally, hepatocytes is under the influence of hepatocyte nuclear factor 4 (HNF4), TGFβ, notch, and Wnt [108]. The first attempt to generate 3D-vascularized iPSC-derived liver buds was published by Takebe et al. [109]. The authors co-cultivated iPSC-derived endoderm-like cells with MSCs and human umbilical vein endothelial cells. The outcome of this specific co-cultivation was a 3D liver bud, which was subsequently transplanted into an immunodeficient mouse to facilitate additional maturation of the bud’s cells into the mature hepatocytes. Later on, Wu et al. successfully generated human iPSC-derived hepatobiliary organoids without any exogenous cells [110]. The three-stage differentiation protocol was based on recapitulating critical aspects of early hepatogenesis via cultivating iPSCs in a medium supplemented by activin A and BMP4 to induce mesodermal and endodermal differentiation and a medium containing FGF4, BMP2, HGF, and keratinocyte growth factor (KGF) for further hepatic progenitors and hepatoblasts differentiation. In the third step, the hepatocyte and cholangiocyte maturation, followed by organoid formation, were under the control of oncostatin M (OSM), cholesterol + MIX, and dexamethasone (Figure 4). In the last few years, liver organoid differentiation methods have advanced rapidly, enabling the generation of 3D-bioprinted liver organoids or liver-on-a-chip [111,112,113]. To date, liver organoids have been successfully used to model various diseases affecting liver tissue and screen novel drug candidates [114,115,116]; therefore, it was not surprising that the research community reached out to them at the beginning of the pandemic to examine pathological processes in the liver caused by SARS-CoV-2 [105,106,117].

Yang et al. reported that human iPSC-derived liver organoids generated by cultivation of iPSCs in Matrigel and sequentially exposed to activin A, BMP4, bFGF, HGF, and OSM, respectively, are permissive to SARS-CoV-2 pseudo-entry virus infection and express strong induction of inflammatory cytokines and chemokines [106]. Another exciting study employing human iPSC-derived liver organoids as a model system for SARS-CoV-2 was published by Richards et al. [105]. The authors utilized a single-cell sequencing technology to identify the cell-intrinsic responses of hepatocytes and cholangiocyte-like cells to SARS-CoV-2. The single-cell RNA sequencing was performed 48 h post-infection. Three major cell clusters were identified, including cholangiocyte-like cells expressing high levels of epithelial cell markers (KRT17, KRT7, and EPCAM), hepatocyte-like cells 1 expressing hepatocyte-specific markers (HNF4, ATF5, and RBP4), and hepatocyte-like cells expressing markers specific for both epithelial cells and hepatocytes representing cells in the de-differentiation state. In liver organoids exposed to live SARS-CoV-2, infected cells were detected among all three cell populations. According to the performed gene enrichment analysis, the authors found that viral infection promotes rapid changes in gene expressions in these cell populations, predominantly affecting inflammatory signaling pathways, such as interferon and IL-6 signaling, which upregulates macrophage MCP-1 expression. Moreover, the most potent induction of inflammatory signaling was observed in hepatocyte-like cells.

The studies mentioned above provide the proof of concept that iPSC-derived liver organoids could serve for COVID-19 modeling involving the investigation of virus tropism and pathogenesis, as well as the facilitation of novel drug discovery. The results of different studies suggest that liver organoids could recapitulate and characterize liver pathology and prove that virus infection has a direct cytopathogenic effect on hepatocytes and cholangiocytes expressing ACE2 and TMPRSS2. Moreover, thanks to single-cell RNA sequencing, it is possible to recognize the cell-intrinsic responses of liver cells to viral infection and, thus, reveal inflammatory pathways, which are liable for hepatitis typical for severe COVID-19. Nevertheless, it is essential to note that iPSC-derived liver organoids likely contain cells at earlier stages of differentiation and, thus, may not fully recapitulate the infection process present in adult tissue.

### 2.5. iPSC-Derived Cardiac and Blood Vessel Organoids

**Figure 5:**
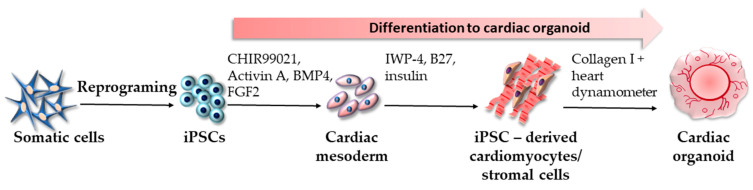
Generation of cardiac organoids according to the protocol by Mills et al. [118].

More than two decades of knowledge in cardiac tissue engineering combined with state-of-the-art iPSC technology led to the development of protocols for manufacturing iPSC-derived cardiac organoids. Mills et al. seeded iPSCs in Matrigel-coated flasks and cultured them for four days in mTeSR-1 [118]. Differentiation into cardiac mesoderm was performed using RPMI B27 medium containing BMP-4, activin A, FGF-2, and CHIR99021. The specification of the iPSC-CMs/stromal cell mixture was achieved by supplementing RPMI B27 with IWP-4. The subsequent flow cytometric analysis revealed that the generated cells were 70% iPSC-CMs and 30% CD90+ stromal cells. Afterward, the cells were mixed with collagen I and pipetted onto the heart dynamometer (Heart-Dyno), facilitating tissue self-formation and eventually establishing the cardiac organoid (Figure 5). In the following years, iPSC-derived cardiac organoids were manufactured using various modified or completely new protocols. Lee et al. reported that the generation of iPSC-derived cardiac organoids could be improved by aurora kinase inhibitor ZM447439 and used for modeling drug safety and efficacy [119]. iPSC-derived cardiac organoids could also be useful for modeling congenital heart anomalies [120], myocardial infarction [121], and other conditions. The newest study reported a protocol that manually induced cell aggregation, leading to a more homogeneous cell population with a suitable size optimal for organoid fabrication [122]. The sudden emergence of the COVID-19 pandemic made iPSC-derived cardiac organoid technology precious as a tool for its modeling since the cardiovascular system is one of many affected by the disease. However, the researchers questioned whether the virus directly damages the heart and blood vessels, cardiovascular impairment is the indirect result of the systemic inflammatory response, or a combination of both scenarios. The imperative need to elucidate these involvements comes from the fact that one of the most dreaded complications of COVID-19, which can affect any organ, is thrombotic microangiopathy [123]. To model blood vessel damage in COVID-19, Monteil et al. fabricated iPSC-derived blood vessel organoids based on the authors’ previous protocol for studying diabetic vasculopathy [124]. iPSCs were resuspended in differentiation media, including Y-27632, and plated for cell aggregation. Afterward, the aggregates were treated with CHIR99021 on day 3, and then BMP4, VEGF-A, and FGF-2 were added on days 5, 7, and 9, respectively. On day 11, to increase endothelial yield and diminish undue pericyte differentiation, the cells were put in a medium supplemented with VEGF-A, FGF-2, and SB43152. The aggregated cells were then embedded in matrigel:collagen I (1:1) gels and further differentiated until the vascular networks were established on day 18. The networks self-assembled into lumenized vascular organoids, closely resembling human capillaries with CR31+ endothelial cells, PDGFR+ pericytes, and the basement membrane. Vascular organoids were then infected with SARS-CoV-2 and analyzed by qRT-PCR for viral detection. The results showed that the virus could readily infect capillaries. The authors also reported that the infection could be significantly hindered by applying the human-recombinant soluble ACE2. Unfortunately, few studies use iPSC-derived cardiac organoids, or any cardiac organoids for that matter, as models of SARS-CoV-2-induced cardiac impairment. Most experiments focused on 2D cultures of iPSC-derived cardiomyocytes, endothelial cells, and other populations of heart cells. For example, Pérez-Bermejo et al. used iPSC-derived cardiac cells and revealed that the virus infects cardiomyocytes, excluding endothelial cells and cardiac fibroblasts. The authors also revealed that the virus induces changes in gene transcription associated with contractility and structural integrity of the sarcomere, indicating that SARS-CoV-2 can directly compromise cardiac inotropy [125]. The hypothesis of direct SARS-CoV-2 damage to cardiomyocytes was reported by yet another 2D model of iPSC-derived cardiomyocytes. The virus was a potent inducer of electrophysiological abnormalities, contractile dysfunction, and even cell death [3]. Although 2D cultures are considered inferior compared to 3D organoids, iPSC-derived cardiomyocytes can still be relevant for drug testing. Choi et al. tested the antiviral activity and safety of the new, promising, antiviral medication—remdesivir. The results showed that while iPSC-derived cardiomyocytes reacted well to the drug regarding its antiviral activity, remdesivir elicited moderate cardiotoxicity, which presented as electrocardiographic abnormalities. Such results led to better drug safety profiling, dosage refinement, and decreased risks of complications, mainly in patients with preexisting heart conditions [126]. In the most recent article, Arhontoulis et al. modeled a COVID-19-associated cytokine storm and its cardiac-damaging potential by fabricating cardiac organoids using iPSC-derived cardiomyocytes (55%), human cardiac ventricular fibroblasts (24%) cultured in FGM-2 medium, human umbilical vein endothelial cells (14%) cultured in EGM-3 medium, and human adipose-derived stem cells (7%). After adding the cell suspension to agarose molds, the organoids formed after four days. Afterward, the organoids were treated with IL-1β—the signature COVID-19 cytokine. As a result, the organoid showed signs of fibrosis and reduced functions. Moreover, the vascularity of IL-1β-treated organoids exhibited prothrombotic features, including the upregulated expressions of ICAM1, E-selectin, and VAP1. On the other hand, NOS3 expression was downregulated, leading to impaired nitric oxide-mediated endothelial functions [127].

Overall, iPSC-derived cardiac organoids represent a great way of disease modeling, including COVID-19, providing insights into the disease pathogenesis, its molecular mechanisms, drug safety, efficacy, and other valuable information. Unfortunately, the fabrication of cardiac organoids is significantly lagging, mainly because of the complex heart structure and its peculiar electrophysiology, which are challenging to reproduce in vitro. The greatest challenges to be addressed in future research are the suboptimal functional and morphological maturities of the organoids. They are the main obstacles preventing the achievement of the most faithful likeness to the heart in vivo. The refinement of approaches to proper vascularization and nutrient distribution is also vital for larger cardiac organoids, which will be the most accurate representation of the heart during both normal and pathological conditions.

### 2.6. iPSC-Derived Kidney Organoids

Acute kidney failure (AKI) is reported to be among the most frequent types of tissue damage in SARS-CoV-2 patients, presented in 37–57% of COVID-19 cases [128,129]. Still, the pathophysiological mechanism of kidney damage has yet to be thoroughly investigated [130].

**Figure 6:**
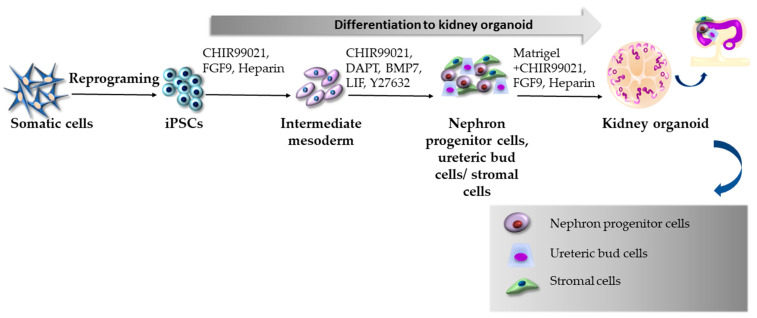
Generation of kidney organoids according to the protocol by Vanslambrouck et al. [133].

To date, few research groups have employed kidney organoids derived from human kidney proximal tubule epithelial cells [131] or ESCs/iPSCs [33,132,133,134] when studying and uncovering the impacts of SARS-CoV-2. The first iPSC-derived kidney organoids, similar to the human fetal kidney, comprising individual nephron segments, including proximal and distal tubules, loops of Henle, and vascularized glomeruli with podocytes, were generated by Takasato et al. [135]. The authors achieved this multicellular kidney organoid differentiation by carefully balancing anterior–posterior patterning of the intermediate mesoderm with small molecules, such as CHIR99021, FGF9, and heparin. Improved iPSC-derived kidney organoids (Figure 6) with enhanced specifications to metanephric nephron progenitors were described by Vanslambrouck et al. [133]. The authors extended the duration of mesodermal patterning while enhancing nephron progenitor expansion, forming strongly proximalized and elongated nephrons displaying distinct segmentations into convoluted and straight segments. Moreover, the suitability of enhanced kidney organoids as a model of SARS-CoV-2 infection and pathogenesis was also examined. A comprehensive analysis of single-cell RNA sequencing (scRNAseq) data displayed expression levels and cellular localization of various entry factors with the prevalence of ACE2 and TMPRSS2 predominantly across the proximal, distal, and endothelial parts but not in podocytes. Infected kidney organoids were analyzed by immunofluorescence for double-stranded RNA (dsRNA) and nephron-specific markers. The results showed the presence of dsRNA mainly in proximal tubules and some detection in loops of Henle. These results are contrary to other studies reporting the presence of SARS-CoV-2 in podocytes [33,136]. The authors hypothesized that the reason might lie in the possible immaturity of nephrons in previously generated kidney organoids. Interestingly, the infected tubular epithelium retained its essential characteristics, but the upregulation of kidney injury marker-1 (KIM-1), a marker sensitive to the early detection of proximal tubule injury, was significantly higher than in the control. However, this enhanced protocol still faces some limitations regarding the nephron pattern (reduced maturity of distal tubule segments) and the presence of off-target populations, such as pre-cartilage cells as a side effect of prolonged BMP signaling. In another research work, Jansen et al. [33] generated iPSC-derived kidney organoids based on Takasato’s protocol with slight modifications to infect them with SARS-CoV-2; they observed the direct effects of the virus on cells within organoids. Certain kidney organoid cellular populations were differentially distributed, imitating the adult kidney, but endothelial progenitors were still clustered in the mesenchyme, which was considered a limitation. In situ hybridization confirmed the expression of the virus in infected podocytes and epithelial cells of proximal tubules, and immune-based correlative light and electron microscopy detected the presence of viral particles within vacuoles in close proximity to the nuclei of stromal cells. According to scRNAseq, the virus caused cell injury and de-differentiation of infected cells with the activation of profibrotic signaling pathways. Furthermore, the infection increased collagen type 1 protein expression, referring to post-infection fibrosis, which was also proved by Masson´s trichrome quantification. In the next step, the authors tested a noncovalent inhibitor of the SARS-CoV-2 main protease (MAT-POS-b3e365b9-1) as a possible prevention of viral uptake by kidney epithelial cells. They found that this inhibitor effectively reduced intracellular viral RNA levels. Based on these results, the authors suggested that the mentioned protease inhibitor might be used to mitigate SARS-CoV-2 viral replication in kidney cells. Garreta et al. developed diabetic kidney organoids derived from ESCs/iPSCs to further expand the relationship between diabetes and SARS-CoV-2 [134]. The authors achieved phenotypic, transcriptional, and metabolic alternations of kidney organoids similar to diabetic kidneys by establishing a cultivation procedure based on high glucose oscillation. The iPSCs were differentiated into kidney organoids with glomerular-like and renal-tubular-like structures via gradual control of CHIR99021, FGF9, heparin, and activin A. Further TEM observations showed that podocyte-like and tubular-like cells exhibited characteristics of late-stage renal differentiation. Interestingly, the authors observed that oscillatory glucose treatment induced a robust ACE2 expression. Diabetic kidney organoids were subsequently infected with SARS-CoV-2 and showed significantly enhanced infection, which was associated with the downregulation of glycolysis-related processes and increased inflammation.

Taken together, iPSC-derived kidney organoids still lack the adult anatomy and more likely resemble second-trimester fetal kidneys. Therefore, the usefulness of such organoids in COVID-19 modeling relies on nephron maturation and their functionality, and researchers must interpret the results of disease modeling in this context. Despite this knowledge, kidney organoids represent a significant advantage in the modeling of tissue damage response following SARS-CoV-2 infection and provide insights into cellular signaling and the contribution of other factors that potentially lead to acute kidney failure.

### 2.7. iPSC-Derived Inner Ear Organoids

**Figure 7:**
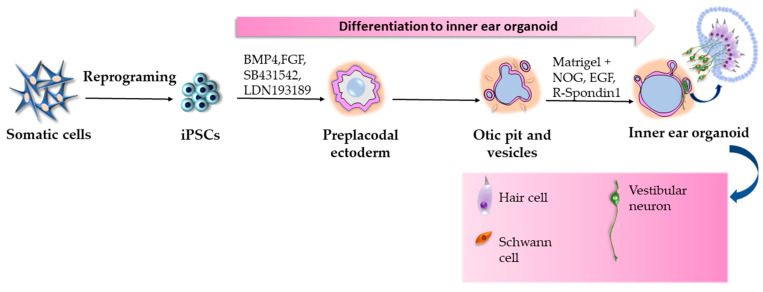
Generation of inner ear organoids according to the protocol by Koehler et al. [141].

It is known that SARS-CoV-2 may also affect the sensory system, often causing anosmia and ageusia [137]. However, reports from COVID-19 patients with audiovestibular symptoms, such as sensorineural hearing loss (SNHL), vertigo, and tinnitus are increasing over time [138,139,140]. The interest in how the virus affects the inner ear is growing, but little is known about its etiopathogenesis. Although, thanks to the generation of 3D inner ear organoids, it is possible to investigate the causal relationship between SARS-CoV-2 and audiovestibular dysfunctions. Among the first authors who reported a method for differentiating human pluripotent stem cells to inner ear organoids with functional hair cells (Figure 7) was Koehler et al. [141]. The authors modulated TGF, BMP, FGF, and WNT signaling in 3D culture systems using small molecules and recombinant proteins (BMP4, FGF-2, CHIR99021, LDN-193189, and SB431542) to generate multiple otic vesicle-like structures from stem cell aggregate. For two months, the otic vesicle-like structures developed into multi-chambered inner ear organoids with non-sensory and sensory epithelia innervated by sensory neurons. Based on this protocol and with the addition of Y-27632 and valproic acid, Jeong et al. generated human iPSC-derived inner ear organoids and infected them with SARS-CoV-2 to investigate whether audiovestibular symptoms are direct consequences of the infection [140]. Notably, the generated inner ear organoids had similar expression patterns of the ACE2 protein to adult human vestibular tissue. At the same time, ACE2 was detected in MYO7A+ hair cell-like cells but not in TUBB3+ axons of neurons innervating hair cell-like cells. However, neuronal cell bodies expressed ACE2. Infected organoids were analyzed by immunostaining for dsRNA, whereas the obtained results showed that the virus targeted vestibular hair cell-like cells. This finding may explain the presence of audiovestibular dysfunctions in COVID-19 patients.

In summary, inner ear organoids represent a suitable approach for modeling the mechanisms of audiovestibular impairment in COVID-19 patients. However, similar to other organoids, several challenges require further investigation. One of the biggest obstacles in inner ear organoid generation is reproducing complex interactions between the cell lineages from which more than 50 inner ear cell types differentiate. To do so, a great bulk of basic embryological research has to be carried out to identify, understand, and induce the molecular pathways vital for normal inner ear development.

## 3. Conclusions and Future Perspectives

Apart from the iPSC-derived organoids discussed in this paper, other organoids have been developed for the purpose of COVID-19 modeling, for instance, organoids generated from tonsillar epithelial cells. Kim et al. demonstrated their expression of key SARS-CoV-2 entry molecules—ACE2, TMPRSS2, and furin, enabling organoid infection and robust replication efficiency. These results prove that tonsil organoids can also serve as suitable ex vivo models of SARS-CoV-2 infection [142].

Without a doubt, iPSC-derived organoids as engineered cell-based models can recapitulate relevant physiological structures and functions. In these uncertain pandemic times, organoid technology has proven to be a valuable and potent platform for investigating the effects of SARS-CoV-2 in different tissues and for screening new drug therapeutics (Table 1). These iPSC–derived organoid models are suitable for using the newest genetic tools, such as single-cell RNA sequencing, by which it is possible to screen for cell types that are positive for the SARS-CoV-2 receptor ACE2 and effector protease TMPRSS2.

Despite the iPSC-derived organoid advantages, such as their availability, editability, and unlimited proliferation capacity, they still possess several disadvantages. The major one is the presence of fetal or neonatal cells/tissues; therefore, additional improvements of differentiation protocols to achieve iPSC-derived organoid maturity need to be done. Another challenge involves further optimization of the protocols for studying infectious diseases, such as COVID-19, by improving organoid complexity and adding immune cells or cells to the vascular system, which are essential to many aspects of COVID-19 pathophysiology. The future development of more advanced organ models represents a combination of 3D printing and organ-on-a-chip technology, by which it will be possible to generate even more physiologically comparable organoids to adult organs. For example, 3D bioprinting allows the creation of macro-scale organoids from tissue-specific cell types laden in bioinks with appropriate spatial cellular locations, such as lung-like organoids providing air–cell surface interference in a complex hollow structure [143].

The organ-on-a-chip technology represents a microfluidic device for cell and tissue culture in continuously perfused small chambers, which enables the study of host–virus interaction, viral pathogenesis, and the development of new antiviral therapeutics in a dynamic and controllable microenvironment [36,144].

Taken together, the above-mentioned studies have contributed enormously to COVID-19 modeling and antiviral drug testing. Despite several limitations, we can conclude that iPSC-derived organoids and organ-on-a-chip systems strengthened by current technologies, such as omics, artificial intelligence, and bioinformatics databases, will gradually replace the current use of animal models and will provide personalized treatment opportunities for various diseases.

## Figures and Tables

**Table 1:** Summary of selected iPSC-derived organoids for COVID-19 modeling.

iPSC-Derived Organoids	Small Molecules, Growth Factors, and Recombinant Proteins Used for Organoid Differentiation	Permissiveness to SARS-CoV-2	Pathological Features	Drug Screening	Ref.
Lung organoids	Activin A, SB431542, Noggin, SAG, FGF4, CHIR99021—foregut endoderm + FGF7, FGF10, CHIR99021, EGF, ATRA, and VEGF/PIGF—lung organoids	Yes	Upregulated expressions of immune-regulatory genes, inflammation-activated inflammasome pathway	EK1 and camostat-inhibition of viral infection	[57]
Lung organoids	CHIR99021, rhKGF, dexamethasone, 8-Br-cAMP, 3-Isobutyl-1-methylxanthine—alveospheres	Yes	Features of cellular damage—alveolar cell hyperplasia with hyaline deposition	15033-7 and DPP4 peptide-inhibition of viral infection	[58]
Brain and choroid plexus organoids	L 2-mercaptoethanol, dorsomorphin, A83-01, CHIR99021, SB431542, insulin, LDN-193189, SHH, and FGF8	Yes—in choroid plexus cells; rarely in neurons and astrocytes	Proinflammatory cytokine response, cell death, formation of syncytia, damage of choroid plexus organoid integrity	-	[76]
BrainSphere organotypic model	BDNF protein, GDNF protein, dorsomorphin, heparin, and cyclopamine	Yes—few neurons	-	-	[77]
Brain and choroid plexus organoids	Heparin, Y27632, L 2-mercaptoethanol, insulin, and vitamin A, BMP4, and CHIR99021	Yes—in choroid plexus surface lining cells	Damage of choroid plexus organoid integrity	-	[78]
Brain organoids	SB431542, dorsomorphin, Y27632, bFGF, EGF, BDNF protein, GDNF protein, L-ascorbic acid, and dibutyryl-cAMP	Yes	Cell death of cortical neurons, reduced number of excitatory synapses	Sofosbuvir—reduction of intracellular viral RNA levels	[79]
Brain organoids	Heparin, Y27632, L 2-mercaptoethanol, insulin, and vitamin A	Yes—only neurons	Presence of aberrant Tau protein in SARS-CoV-2 + neurons	-	[81]
Brain organoids	Heparin, Y27632, L 2-mercaptoethanol, insulin, and vitamin A	Yes—only neurons	Cell death of infected and neighboring cells	-	[82]
3D neurospheres	Y27632, dorsomorphin, SB431542, FGF2, BDNF protein, GDNF protein, β-mercaptoethanol, and L-ascorbic acid	Yes—neural progenitor cells	Cell death	-	[83]
Enteroids Colonoids	Dorsomorphin, SB431542, CHIR99021, rhBMP4, and retinoic acid-definitive endoderm + Y27632—final organoid	Yes—in enterocytes and colonocytes	Upregulation of apoptosis-related genes, cellular stress, expression of inflammatory markers	-	[86]
Liver organoids	Activin A, BMP4, FGF4, CHIR9902, retinoic acid, HGF, and OSM	Yes—more in hepatocytes-like cells, less in cholangiocytes-like cells	Rapid change of gene expression in inflammatory signaling pathways	-	[105]
Liver organoids	Activin A, BMP4, bFGF, HGF, and OSM	Yes	Strong induction of inflammatory cytokines and chemokines	-	[106]
Blood vessel organoids	Y-27632, CHIR99021, BMP4, VEGF-A, FGF-2, and SB43152	Yes	-	Human recombinant soluble ACE2-inhibition of viral infection	[124]
Cardiac organoids	Self-assembly process	Yes	Signs of fibrosis and reduced functions of cardiomyocytes, prothrombotic features of the vasculature–impaired nitric oxide-mediated endothelial function	-	[127]
Kidney organoids	CHIR99021, FGF9, and heparin	Yes—mainly in proximal tubules, less in loops of Henle	Upregulation of KIM-1 in proximal tubules	-	[133]
Kidney organoids	CHIR99021, FGF9, hEGF, and heparin	Yes—in podocytes and proximal tubules	Cell injury and dedifferentiation of infected cells, post-infection fibrosis	MAT-POS-b3e365b9-1—reduction of intracellular viral RNA levels	[33]
Diabetic kidney organoids	CHIR99021, FGF9, heparin, and activin A	Yes	Downregulation of glycolysis-related processes and increased inflammation	-	[134]
Inner ear organoids	BMP4, FGF-2, CHIR99021, LDN-193189, SB431542, and Y-27632	Yes—vestibular hair cell-like cells	dsRNA presented in vestibular hair cell-like cells	-	[140]

## Data Availability

Not applicable.
